# Bacterial contamination of dental unit waterlines

**DOI:** 10.1007/s10661-012-2812-9

**Published:** 2012-08-17

**Authors:** Jolanta Szymańska, Jolanta Sitkowska

**Affiliations:** 1 Chair and Department of Paedodontics, Medical University, Karmelicka 7, 20-018 Lublin, Poland; 2 Department of Occupational Biohazards, Institute of Agricultural Medicine, Jaczewskiego 2, 20-090 Lublin, Poland

**Keywords:** Dental unit waterlines, Bacterial contamination, Aerobic and facultative anaerobic bacteria

## Abstract

Safety of patients and dental personnel requires the appropriate microbiological water quality in dental units. During treatment, patients and dental workers are exposed both to direct contact with bacteria-contaminated water in the form of splatter and with contaminated water aerosol emitted during work by unit handpieces, including rotating and ultrasonic instruments. The aim of the study was to determine the qualitative and quantitative contamination of water in dental unit reservoirs with aerobic and facultative anaerobic bacteria. The study material included water sampled from 107 dental unit reservoirs located in dental surgeries of public health centres. Conventional microbiological methods were used to identify microorganisms. The study shows that the contamination of water in dental unit reservoirs with aerobic and facultative anaerobic bacteria is commonplace. The mean concentration of mesophile bacteria in dental unit reservoir water exceeded 1.1 × 10^5^ cfu/ml. The prevailing species were Gram-negative bacteria of the families Burkholderiaceae, Pseudomonadaceae, Ralstoniaceae and Sphingomonadaceae. The most numerous bacteria were *Ralstonia pickettii*, constituting 49.33 % of all the identified aerobic and facultative anaerobic bacteria. Among Gram-positive rods, the most numerous were bacteria of the genus *Brevibacterium* (5.83 %), while the highest percentage shares (13.25 %) of all Gram-positive microorganisms were found for *Actinomyces* spp. The study confirms the necessity of regular monitoring of microbial contamination of dental unit waterlines (DUWL) and use of various water treatment procedures available to disinfect DWUL.

## Introduction

Safety of dental patients and dental personnel requires an appropriate microbiological quality of water used in dental units. Flowing from working handpieces, water cools dental equipment and rinses oral tissues. During dental treatment, patients and personnel are exposed both to direct contact with bacteria-contaminated water in the form of splatter and with contaminated water aerosol emitted during work by unit handpieces, including rotating and ultrasonic instruments (Kumar et al. 2010). A high level of microbial contamination, presence of opportunistic microorganisms and bacterial endotoxin associated with Gram-negative bacteria are the most important health risk factors transmitted by water from dental units (Szymańska et al. 2008; Coleman et al. 2009; Singh and Mabe 2009; Pankhurst and Coulter 2007). Microbial composition of water exiting from unit of working handpieces depends on the microbiological quality of water flowing into a unit, but also, as many researchers stress, by the biofilm present on the walls of tubing that constitutes dental unit waterlines (DUWL) (O'Donell et al. 2011; Kumar et al. 2010). Dental units with open water systems are provided with water from public water supplies, while for the units with closed systems, water is from a built-in reservoir. It is interesting to determine the microbial quality of water in unit reservoirs. The aim of the study was to determine the qualitative and quantitative contamination of water in dental unit reservoirs with aerobic and facultative anaerobic bacteria.

## Material and methods

The study material included water sampled from 107 dental unit reservoirs located in dental surgeries of public health centres in the Lubelskie Voivodship, Poland. In order to guarantee identical sampling conditions and to avoid accidental microbiological contamination of water, all samples were taken successively in winter (heating season), at the beginning of a working day and before patient consultations were started. Water samples were placed in sterile, airtight test tubes. Considering the scientific character of the study, as well as the necessity to warrant anonymity (protection of data on health centres), the method of double coding of samples was used. An agreement of the owner of a surgery or the director of the health centre was obtained before each sampling

## Microbiological examination of water samples

In order to isolate and identify microorganisms, conventional microbiological methods were used. Mesophile Gram-positive and Gram-negative bacteria with increased nutrition requirements were cultured on nutrient agar with 5 % sheep blood. Eosin methyl blue agar (EMB) was used for isolation and initial identification of Gram-negative rods. The examined samples were inoculated on both media simultaneously, using the plate dilution method with surface inoculation. The initial water samples (0.1 ml) and their tenfold dilutions in sterile physiological salt solution (0.85 % NaCl) were introduced twice, parallelly, to each of the two media and distributed evenly on the agar surface with a sterile glass spreader. The water inoculations on blood and EMB agar were incubated for 24 h at 35–37 °C and next for 3 days at room temperature (22 °C) and 3 days at refrigerator temperature (4 °C). The prolonged culture at low temperature favoured the growth of some of mesophile and psychrophile microorganisms. After incubation, the initial identification of microorganisms cultured on both media was performed. The assessment of the growth of bacterial colonies on the media included their macroscopic morphological characteristics, such as the size and form of colonies, surface and margin, colour, opacity and texture. Microscopic preparations were made from the colonies differing in appearance with the use of Gram staining methods. The analysis of their microscopic image estimated the colour of bacterial cell staining, shape, size, arrangement of the neighbouring cells and the presence of spores. Next, considering the previously described characteristics, the number of morphological types was determined, as well as their concentration, expressed in colony-forming units in 1 ml of water (cfu/ml) according to the formula:

$$ x = a \times r/0.1 $$

where

*x*: the concentration of bacteria in water expressed with the number of colony-forming units in 1 ml water
*a*: the average number of colonies on a plate
*r*: the reverse of the dilution

In order to obtain a reliable number of bacterial colonies on the plates, the count was performed when 100–300 microorganisms were present.

Subsequently, bacterial colonies more frequently occurring in inoculations on each of the media were isolated and identified down to the level of genus or species, using biochemical microtests. Gram-negative rods from EMB agar were identified with API 20E and API 20NE tests (bioMeriéux, Marcy l’Etoile, France), while Gram-positive bacteria from blood agar were identified with GP2 MicroPlate™ test (BIOLOG, Inc., Hayward, USA). Gram-negative rods impossible to determine with the API kit were identified with the analogous test GP2 MicroPlate™. All the tests were used according to the procedures recommended by the manufacturers.

## API test technique

The initial identification of aerobic Gram-negative rods was performed by testing the ability to produce cytochrome oxidase by the examined strains. The bacterial mass cultured within 24 h was applied onto the reactive surface of the test strip (Bactident Oxidase, Merck, Germany), and after 20–60 s, the result was read. Blue or purple–blue colour of the strip indicated an oxidase-positive strain; the absence of colour indicated an oxidase-negative one. Oxidase-positive strains were identified with API 20NE test and the oxidase-negative ones with API 20E. The strips of both API tests consisting of 20 microtubes containing dehydrated substrates were filled, according to the manufacturer's manual, with the previously prepared bacterial suspension, of an appropriate density, in sterile physiological liquid (in some microtubes, anaerobic conditions were created by covering their surface with liquid sterile paraffin). The strips were placed in humid chambers and incubated—according to the API kit used—for 24 h at 35–37 °C (in the case of API 20E) and 24–48 h at 30 °C (in the case of API 20NE). The final and specific results of API 20NE were read after full 48 h. Metabolic processes during incubation caused colour changes in microtubes—spontaneously or due to added reagents. The results of those reactions in the form of seven-digit numeric code were used to identify the examined strain.

## GP2 and GN2 MicroPlate™ test technique

The system Biolog—GP2 and GN2—MicroPlate™ is a standardized micromethod used to identify aerobic and facultative anaerobic Gram-positive and Gram-negative bacteria on the basis of their metabolic pattern. The test determines the capacity of microorganisms to biochemical reactions with substrates contained in reaction wells. The suspension (18 ml) of the strains selected for identification in gelled 0.40 % NaCl was prepared; the appropriate cell density, different for Gram-negative and Gram-positive bacteria, was determined with a turbimeter.

Of the suspension, 150 μl was added to each of 96 wells in the reactive plate (one of them was a negative control, containing only indicator substance). Subsequently, the microplates were incubated for 24 h at 30 or 35 °C, according to the microorganisms to be determined. The colour indicator in each of the wells—tetrazolium violet—as a result of the reaction responded with the change of colour into purple in the positive wells containing a given strain. Results were read after 6 and 24 h, comparing the colour of liquid in individual wells with the negative control. The final identification was made with the MicroLog™ software, provided by the manufacturer, determining the degree of conformity and probability for an identified microorganism.

## Results and discussion

### Qualitative assessment of dental unit reservoir water

In all 107 tested water samples, mesophile bacteria were found. Among them, Gram-negative rods were the largest group including the following species: *A* *cidovorax avenae* ss *cattleyae*, *Alcaligenes faecalis*, *Brevundimonas vesicularis*, *Burkholderia cepacia*, *Pseudomonas aeruginosa*, *Pseudomonas chlororaphis*, *Pseudomonas fluorescens*, *Pseudomonas huttiensis* (*Burkholderia*-like), *Pseudomonas putida*, *Pseudomonas stutzeri*, *Pseudomonas syringae* pv. *aptata*, *Ralstonia pickettii*, *Sphingomonas paucimobilis*, *S. paucimobilis* B, *Sphingobacterium spiritovorum* and *Stenotrophomonas maltophilia*. *R. pickettii* was identified in 52 workstations, which constituted 48.6 % of all the studied units. The second most numerous, in respect of the number of samples, were Gram-negative rods, represented by *Sphingomonas paucimobilis* and *P. fluorescens*, 27 (25.23 %) and 16 (14.95 %) workstations, respectively. The species identified in individual units (0.93 %) included: *A. avenae* ss *cattleyae*, *P. aeruginosa*, *B. cepacia*, *P. huttiensis* (*Burkholderia*-like), *P. syringae* pv. *aptata* and *S. paucimobilis* B. They occurred in 5.61 % of all the studied units and 6.59 % of the workstations where Gram-negative rods were isolated (Table 1).

**Table 1:** The presence of bacteria in water sampled from dental units

Genus/species	Number of units	Percentage share
Gram-negative rods		
* Acidovorax avenae* ss *cattleyae*	1	0.93
* Alcaligenes faecalis*	3	2.8
* Brevundimonas vesicularis*	2	1.87
* Burkholderia cepacia*	1	0.93
* Pseudomonas syringae* pv. *aptata*	1	0.93
* Pseudomonas aeruginosa*	1	0.93
* Pseudomonas chlororaphis*	3	2.8
* Pseudomonas fluorescens*	16	14.95
* Pseudomonas huttiensis* (*Burkholderia*-like)	1	0.93
* Pseudomonas putida*	4	3.74
* Pseudomonas stutzeri*	2	1.87
* Ralstonia pickettii*	52	48.6
* Sphingobacterium spiritovorum*	3	2.8
* Sphingomonas paucimobilis*	27	25.23
* Sphingomonas paucimobilis* B	1	0.93
* Stenotrophomonas maltophilia*	4	3.74
Gram-positive rods		
* Arthrobacter histidinolovorans*	1	0.93
* Arthrobacter ilicis*	1	0.93
* Arthrobacter* spp.	1	0.93
* Arthrobacter woluwensis*	1	0.93
* Aureobacterium* spp.	2	1.87
* Brevibacterium epidermidis*	5	4.67
* Brevibacterium mcbrellneri*	1	0.93
* Brevibacterium otitidis*	1	0.93
* Brevibacterium* spp.	4	3.74
* Brevibacterium* spp. (CDC. B-1/3)	2	1.87
* Clavibacter michiganensis* ss *insidiosus*	1	0.93
* Corynebacterium auris*	2	1.87
* Corynebacterium* spp.	7	6.54
* Corynebacterium urealyticum*	2	1.87
* Corynebacterium variabile*	1	0.93
Other Gram-positive rods	5	4.67
* Microbacterium flavescens*	2	1.87
* Microbacterium laevaniformans*	8	7.48
* Microbacterium* spp.	5	4.67
* Microbacterium* spp. (CDC. A-5)	5	4.67
* Microbacterium testaceum*	1	0.93
* Rhodococcus fascians*	1	0.93
* Rhodococcus* spp.	1	0.93
Gram-positive cocci		
* Enterococcus casseliflavus*	1	0.93
* Micrococcus* spp.	20	18.69
* Pediococcus pentosaceus*	1	0.93
* Staphylococcus arlettae*	1	0.93
* Staphylococcus haemolyticus*	1	0.93
* Staphylococcus lentu*	1	0.93
* Staphylococcus lugdunensis*	1	0.93
* Staphylococcus saprophyticus*	2	1.87
* Staphylococcus sciuri* ss *rodentium*	1	0.93
* Staphylococcus* spp.	32	29.91
* Stomatococcus mucilaginosus*	4	3.74
* Streptococcus acidominimus*	2	1.87
* Streptococcus* spp.	5	4.67
Spore-forming Gram-positive rods		
* Bacillus halodurans*	3	2.8
* Bacillus* spp.	10	9.34
*Actinomyces*		
* Actinomyces* spp.	21	19.63
* Streptomyces albus*	2	1.87

The isolated Gram-positive rods belonged to the following genera and species: *Arthrobacter histidinolovorans*, *Arthrobacter* spp., *Arthrobacter woluwensis*, *Microbacterium flavescens*, *Aureobacterium* spp., *Microbacterium testaceum*, *Brevibacterium epidermidis*, *Brevibacterium otitidis*, *Brevibacterium* spp., *Brevibacterium* spp. (CDC. B-1/3), *Clavibacter michiganensis* ss *insidiosus*, *Corynebacterium auris*, *Corynebacterium* spp., *Corynebacterium urealyticum*, *Corynebacterium variabile*, *Brevibacterium mcbrellneri*, *Arthrobacter ilicis*, *Microbacterium laevaniformans*, *Microbacterium* spp., *Microbacterium* spp. (CDC. A-5), *Rhodococcus fascians* and *Rhodococcus* spp. Among them, the most frequently found were *M. laevaniformans*, present at eight workstations (7.48 %) and *Corynebacterium* spp. at seven workstations (6.54 %). *B. epidermidis*, *Microbacterium* spp., *Microbacterium* spp. (CDC. A-5) and other unidentified Gram-negative rods occurred at five workstations (4.67 %). *Brevibacterium* spp. was found in four water samples, i.e. in 3.74 % of all the units. The following bacteria were present at individual workstations: *A. histidinolovorans*, *Arthrobacter* spp., *A. woluwensis*, *M. testaceum*, *B. otitidis*, *C. michiganensis* ss *insidiosus*, *C. variabile*, *B. mcbrellneri*, *A. ilicis*, *R. fascians* and *Rhodococcus* spp. (0.93 %; Table 1).

The following Gram-positive cocci were identified: *Enterococcus casseliflavus*, *Micrococcus* spp., *Pediococcus pentosaceus*, *Staphylococcus arlettae*, *Staphylococcus haemolyticus*, *Staphylococcus lentus*, *Staphylococcus lugdunensis*, *Staphylococcus saprophyticus*, *Staphylococcus sciuri* ss *rodentium*, *Staphylococcus* spp., *S* *tomatococcus mucilaginosus*, *Streptococcus acidominimus* and *Streptococcus* spp. At 32 workstations, *Staphylococcus* spp. (29.91 %) was found, and at 20, *Micrococcus* spp. was also found (18.69 %). The species *E. casseliflavus*, *P. pentosaceus*, *S. arlettae*, *S. haemolyticus*, *S. lentus*, *S. lugdunensis* and *S. sciuri* ss *rodentium* were isolated at individual workstations, which constituted 0.93 % of all the water samples (Table 1).

Among spore-forming Gram-positive bacteria, *Bacillus* spp. were found at ten workstations (9.34 %) and *Bacillus halodurans* at three workstations (2.8 %; Table 1). *A* *ctinomyces* spp. were isolated from 21 samples (19.63 %) of the tested unit water and *Streptomyces albus* from two samples (1.87 %; Table 1).

### Quantitative assessment of dental unit reservoir water

Quantitative assessment of particular genera/species of bacteria in water samples showed that the total average concentration of all the bacteria isolated from the reservoirs was 110,165.28 cfu/ml, the minimum concentration was 30.00 cfu/ml and the maximum was 1,234,000.00 cfu/ml. For Gram-negative rods, the average concentration is 71,103.64 cfu/ml, reaching the highest level for *R. pickettii* (53,344.07 cfu/ml) and subsequently for *P. putida* (5,822.94 cfu/ml), *S. paucimobilis* (5,276.82 cfu/ml), *P. chlororaphis* (2,439.25 cfu/ml) and *P. fluorescens* (1,693.83 cfu/ml). Among Gram-negative rods, the lowest concentrations were reached by *B. cepacia* (0.05 cfu/ml), *P. aeruginosa* (0.19 cfu/ml), *P. huttiensis* (*Burkholderia*-like; 0.19 cfu/ml) and *A. avenae* ss *cattleyae* (0.93 cfu/ml). Concentration values for other species of Gram-negative rods varied between 915.89 and 2.90 cfu/ml (Table 2).

**Table 2:** The average concentration of particular genera/species of bacteria isolated from dental unit water samples

Genera/species	cfu/ml
Gram-negative rods	71,103.64
*Acidovorax avenae* ss *cattleyae*	0.93
*Alcaligenes faecalis*	794.63
*Pseudomonas aeruginosa*	0.19
*Burkholderia cepacia*	0.05
*Pseudomonas chlororaphis*	2,439.25
*Pseudomonas fluorescens*	1,693.83
*Pseudomonas huttiensis* (*Burkholderia*-like)	0.19
*Pseudomonas putida*	5,822.94
*Pseudomonas stutzeri*	439.25
*Pseudomonas syringae* pv. *aptata*	915.89
*Brevundimonas vesicularis*	201.40
*Ralstonia pickettii*	54,344.07
*Sphingobacterium spiritovorum*	384.58
*Sphingomonas paucimobilis*	5,276.82
*Sphingomonas paucimobilis* B	238.32
*Stenotrophomonas maltophilia*	2.90
Gram-positive rods	13,262.78
*Arthrobacter histidinolovorans*	1.87
*Arthrobacter ilicis*	70.09
*Arthrobacter* spp.	0.84
*Arthrobacter woluwensis*	0.42
*Aureobacterium* spp.	6.68
*Brevibacterium epidermidis*	1,239.44
*Brevibacterium mcbrellneri*	15.42
*Brevibacterium* spp.	6,424.30
*Brevibacterium* spp. (CDC. B-1/3)	275.70
*Brevibacterium otitidis*	13.41
*Clavibacter michiganensis* ss *insidiosus*	93.46
*Corynebacterium auris*	20.70
*Corynebacterium* spp.	626.26
*Corynebacterium urealyticum*	1,892.52
*Corynebacterium variabile*	11.70
Gram-positive rods	7.24
*Microbacterium flavescens*	62.62
*Microbacterium laevaniformans*	118.27
*Microbacterium* spp.	71.96
*Microbacterium* spp. (CDC. A-5)	57.85
*Microbacterium testaceum*	2.80
*Rhodococcus fascians*	602.80
*Rhodococcus* spp.	200.93
Gram-positive cocci	868.97
*Enterococcus casseliflavus*	14.95
*Micrococcus* spp.	239.44
*Pediococcus pentosaceus*	369.16
*Staphylococcus arlettae*	14.02
*Staphylococcus haemolyticus*	0.56
*Staphylococcus lentus*	0.89
*Staphylococcus lugdunensis*	6.54
*Staphylococcus saprophyticus*	9.21
*Staphylococcus sciuri* ss *rodentium*	0.42
*Staphylococcus* spp.	140.14
*Stomatococcus mucilaginosus*	8.22
*Streptococcus acidominimus*	14.81
*Streptococcus* spp.	50.61
Spore-forming Gram-positive rods	1,185.14
*Bacillus halodurans*	1,144.86
*Bacillus* spp.	301.03
Actinomyces	14,896.07
*Actinomyces* spp.	14,602.34
*Streptomyces albus*	4.72
Average for total bacteria	110,165.28
Min. for a unit	30.00
Max. for a unit	1,234,000.00

The total concentration of all Gram-positive rods was 13,262.78 cfu/ml. The highest values were reached by the following genera and species: *Brevibacterium* spp. (6,424.30 cfu/ml), *C. urealyticum* (1,892.52 cfu/ml) and *B. epidermidis* (1,239.44 cfu/ml) and the lowest by *A. woluwensis* (0.42 cfu/ml) and *Arthrobacter* spp. (0.84 cfu/ml). Concentration values for the remaining Gram-positive rods varied from 626.26 to 1.87 cfu/ml (Table 2).

The total concentration of Gram-positive cocci was 868.97 cfu/ml. The highest cfu/ml values were reached by (cocci) *P. pentosaceus* (369.16 cfu/ml), *Micrococcus* spp. (239.44 cfu/ml) and *Staphylococcus* spp. (140.14 cfu/ml) and the lowest by *S. sciuri* ss *rodentium* (0.42 cfu/ml), *S. haemolyticus* (0.56 cfu/ml) and *S. lentus* (0.89 cfu/ml). The concentrations of other cocci varied from 6.54 to 50.61 cfu/ml (Table 2).

The concentration of spore-forming rods was 1,185.14 cfu/ml, with *B. halodurans* reaching 1,144.86 cfu/ml and *Bacillus* spp. 301.03 cfu/ml (Table 2). The total concentration of *Actinomyces* isolated from the water samples was 14,896.07 cfu/ml, with *Actinomyces* spp. reaching 14,602.34 cfu/ml and *S. albus* 4.72 cfu/ml (Table 2).

Among the isolated Gram-negative rods, *R. pickettii* prevailed in all the samples and constituted 49.33 % of all the bacteria. In a significant number of samples, the species *P. putida* (5.28 %), *S. paucimobilis* (4.79 %), *P. chlororaphis* (2.21 %) and *P. fluorescens* (1.54 %) were present. As a result of rounding the results to two decimal places, the percentage shares of *P. aeruginosa*, *B. cepacia*, *P. huttiensis* and *S. maltophilia* were 0.00 %. The percentage composition of other species of Gram-negative rods varied between 0.08 and 0.83 %.

Among Gram-positive rods, the species *Brevibacterium* (5.83 %), *C. urealyticum* (1.72 %) and *B. epidermidis* (1.12 %) were present in the largest proportions; *A. histidinolovorans*, *Arthrobacter* spp., *A. woluwensis*, *Aureobacterium* spp., *M. testaceum* and other unidentified Gram-positive rods were the least numerous (0.00 %). The percentage composition of other species varied between 0.01 and 0.57 %.

Gram-positive cocci were most numerously represented by the following genera and species: *P. pentosaceus* (0.33 %), *Micrococcus* spp. (0.22 %) and *Staphylococcus* spp. (0.13 %). *S. haemolyticus*, *S. lentus*, *S. lugdunensis*, *S. saprophyticus*, *S. sciuri* ss *rodentium*, *S. mucilaginosus* and *S. acidominimus* had the smallest percentage share. Other cocci reached the percentage composition varying between 0.01 and 0.04 %.

Spore-forming Gram-positive rods isolated from the water samples were represented by: *B. halodurans* (1.04 %) and *Bacillus* spp. (0.27 %). Among the detected actinomyces, the *Actinomyces* spp. reached a high percentage share—13.25 %—while the quantity of *S. albus* was insignificant.

The comparison of the percentage share of particular groups of bacteria isolated from the water samples showed that the most numerous group was Gram-negative rods (70.18 %), followed by *Actinomyces* (14.70 %), Gram-positive (13.09 %) and spore-forming rods (1.17 %) and Gram-positive cocci (0.86 %).

## Discussion

Water for testing was taken from various segments of DUWL; most frequently, it was water flowing from unit handpieces (high-speed microengines, air–water syringes and ultrasonic scalers), less frequently from reservoirs (bottles/containers) of the units. Research shows that that the mean concentration of microorganisms in reservoir water was: >3.9 × 10^4^; 6.6 × 10^4^; 2.01 × 10^5^; or 0–1.52 × 10^6^ cfu/ml (Szymańska et al. 2008). In later studies, the detected concentration reached 3.17 × 10^5^ cfu/ml (Türetgen et al. 2009). Our study found that the mean concentration of aerobic and facultative anaerobic bacteria in water from 107 reservoirs was 1.1 × 10^5^ cfu/ml, which is lower than the results most frequently obtained in other studies. It should be noted that the minimum contamination level detected in our studies was 3.0 × 10^1^ cfu/ml, while the maximum reached 1.23 × 10^6^ cfu/ml—the values similar to those found in the Brazilian research (Souza-Gugelmin et al. 2003). The cited studies found some dental units free from microbial contamination, while in our study, all the units were contaminated.

According to American Dental Association (ADA), dental water should not have more than 200 cfu/ml of aerobic, mesophilic, heterotrophic bacteria, and according the ADA and the Center for Disease Control and Prevention's conclusion, maximum contamination of dental treatment water should be <500 cfu/ml (Lin et al. 2011). Generally, bacterial contamination detected in the unit reservoirs examined by the authors very significantly exceeded the recommended values. This points to the strict necessity of a DUWL decontamination protocol. The advantages of a closed dental unit water system should be used, as well as the possibility to apply different biocides (Liaqat and Sabri 2010; Lin et al. 2011).

Studies in other European countries show that water in dental units in Göteborg is generally not acceptable and does not fulfil drinking water standard. Of the 405 dental units, 303 (75 %) did not have acceptable (<100 cfu/ml fast growing and <500 cfu/ml of slow growing bacteria) water quality (Dahlén et al 2009), and microbiological quality of water from dental units in one of the cantons in Switzerland did not comply with the Swiss drinking water standards nor the recommendations of the American Centers for Control and Prevention (Barben et al. 2009).

In our study, the qualitative bacteriological assessment of dental unit reservoir water showed the prevalence of Gram-negative bacteria from the families Burkholderiaceae, Pseudomonadaceae, Ralstoniaceae and Sphingomonadaceae, which confirms prior research conducted by one of the authors in the same region of Poland. The latter study found that *R. pickettii* was the most numerous bacterial species detected in unit reservoir water, making up 96.5 % of all the isolated aerobic and facultative anaerobic bacteria, with the mean concentration was 1.9 × 10^5^ cfu/ml. Less frequently isolated Gram-negative bacteria were *S. paucimobilis* (1.32 %), with the mean concentration 2.6 × 10^3^ cfu/ml, and *B. vesicularis* (1.07 %), with the mean concentration 2.1 × 10^3^ cfu/ml (Szymańska 2007). The study gave similar results concerning the prevailing bacterial species, but the percentage of *R. pickettii* was much lower (49.33 %; 5.4 × 10^4^ cfu/ml), and *S. paucimobilis* constituted 4.79 % (5.2 × 10^3^ cfu/ml) of all the isolated bacteria. Bacteria of the species *R. pickettii* and *S. paucimobilis* were identified in earlier studies of water in dental unit reservoirs (Barbeau et al. 1996; Meiller et al. 1999; Uzel et al. 2008; Williams et al. 1996).

The present research found that 5.28 % of the total bacteria were *P. putida* (5.8 × 10^3^ cfu/ml), which, as the literature indicates, were isolated only by Barbeau et al (1996). Other authors detected *P. fluorescens* in dental units (Barbeau et al. 1996; Göksay et al. 2008; Uzel et al 2008; Williams et al. 1996), which in our study was 1.54 % of all the isolated bacteria.

Among Gram-positive rods, bacteria of the *Brevibacterium* spp. (6.4 × 10^3^ cfu/ml; 5.83 %) were most numerous; the available literature does not report their presence. Similarly, the authors did not find reports on isolating *C. urealyticum* and *B. epidermidis* from DUWL water. In the present study, their concentrations were, respectively, 1.8 × 10^3^ cfu/ml (1.72 % of total bacteria) and 1.2 × 110^3^ cfu/ml (1.12 % of total bacteria). Those bacteria, characteristic of physiological flora of the human skin and mucosa and present in the environment of a dental surgery, probably entered the unit water reservoir during work (Bennett et al. 2000; Harell and Molinari 2004; Donlan 2002). The fact of isolating in our study *C. michiganensis* ss *insidiosus*—a fitopathogen, alien to Polish microflora, causing bacterial wilt of lucerne (http://www.iop.krakow.pl/ias/Gatunek.aspx?spID=627)—is interesting but difficult to explain. Previous studies show that DUWL contain Gram-positive cocci of the genus *Micrococcus* and *Staphylococcus* spp. (Göksay et al. 2008; Meiller et al. 1999; Pankhurst et al. 1998; Szymańska 2007; Williams et al. 1996), which was confirmed by our research; however, the concentration of those bacteria was low.

Spore-forming Gram-positive rods of the genus *Bacillus* spp. were found in the studies conducted in 1990s; however, the lack of information of the quantitative share of individual species of that genus makes a comparison impossible (Barbeau et al. 1996; Meiller et al. 1999; Pankhurst et al. 1998; Williams et al. 1996). Bacteria of that genus were also isolated in our study; however, their quantity was not significant compared to other microorganisms. Among spore-forming Gram-positive rods of the genus *B. halodurans*, closely related to water environment and soil (Costerton et al. 1995), its mean concentration was 1.1 × 10^3^ cfu/ml, and the percentage share in all microorganisms is 1.04 %.

In the present study, the highest concentration (1.4 × 10^4^ cfu/ml) and percentage share (13.25 %) in all Gram-positive bacteria were found for bacteria of the genus *Actinomyces*. They were isolated from water also in prior studies (Barbeau et al. 1996; Pankhurst et al. 1998).

The results of our own studies, as well as those of other authors conducted over the years, show a high number of microorganisms found in DUWL (Dahlén et al. 2009; Göksay et al. 2008; Walker 2004). An extremely high bacterial level is considered as a particular hazard for certain groups of patients, e.g. immunocompromised or elderly persons, as well as for dental workers exposed to long-lasting influence of microorganisms present in water. Therefore, it is necessary to regularly monitor microbiological quality of water in DUWL, including detection of opportunistic pathogens when bacterial contamination is expected, prevent water stagnation in DUWL and use various treatment procedures available to disinfect DUWL and reduce biofilm development, which is stressed by the researchers studying the problem (Abdallah and Khalil 2011; Aprea et al. 2010; Kumar et al. 2010).

## Conclusions

1. The contamination of water in dental unit reservoirs with aerobic and facultative anaerobic bacteria is commonplace, and the mean concentration exceeds 1.1 × 10^5^ cfu/ml.
2. The most numerous bacteria colonizing water in dental unit reservoirs are Gram-negative rods, Gram-positive cocci, Gram-positive rods and *Actinomyces* are less numerous, and spore-forming Gram-positive rods are the least numerous.
3. Bacteria of the species *R. pickettii* colonize almost half of dental unit reservoirs, reach the highest concentration and constitute half of the total mesophile bacteria contaminating water in dental unit reservoirs.
4. Regular monitoring of microbiological contamination of DUWL water and application of various treatment procedures available to disinfect DUWL are necessary.
